# Guanfacine extended-release use in diagnostic diversity and symptom complexity

**DOI:** 10.3389/frcha.2026.1912093

**Published:** 2026-08-21

**Authors:** Gülenay Korkmaz Şahin, Seda Sarı, Zeynep İrem Şener, Nazlı Burcu Özbaran

**Affiliations:** 1 Department of Child and Adolescent Psychiatry, Ege University Faculty of Medicine, İzmir, Türkiye; 2 Department of Child and Adolescent Psychiatry, Behçet Uz Training and Research Hospital, İzmir, Türkiye

**Keywords:** attention-deficit/hyperactivity disorder, autism spectrum disorder, child and adolescent, guanfacine extended-release, neurodevelopmental disorders

## Abstract

**Objective:**

This retrospective chart review examined clinical changes in ADHD symptoms and global functioning following guanfacine extended-release (GXR) initiation in a diagnostically heterogeneous clinical sample of children and adolescents with ADHD.

**Methods:**

Thirty-eight children and adolescents aged 6–18 years with ADHD, with or without comorbid neurodevelopmental or psychiatric conditions, who were prescribed guanfacine extended-release were included in the tolerability analysis. Pre–post clinical outcome analyses were conducted in 33 patients who continued GXR for at least three months and had follow-up scale assessments available between weeks 4 and 12 after treatment initiation. Clinical data, including ADHD-related rating scales and autism-related clinical measures, were obtained retrospectively from medical records. Diagnoses were based on DSM-5 criteria, and psychiatric comorbidities were assessed using K-SADS-PL.

**Results:**

Of the participants, 76.3% were male. All participants had ADHD, and 63.2% had comorbid ASD. The mean final weight-adjusted GXR dose was 0.05 ± 0.03 mg/kg/day. Adverse effects were reported in 28.9% of patients, most commonly restlessness. Among 33 patients with follow-up assessments obtained between weeks 4 and 12, median CGI-S scores decreased from 5 to 3, while GAS scores increased from 55 to 75. ADHD symptom ratings decreased in most Turgay scale subdomains, particularly attention deficit, hyperactivity/impulsivity, and oppositional defiant symptoms.

**Conclusion:**

Guanfacine extended-release use was associated with improvements in ADHD symptom ratings and global functioning in this retrospective clinical sample. Adverse effects were observed in a clinically relevant subset of patients and led to discontinuation in some cases. Given the retrospective design, lack of a control group, small sample size, and concomitant medication use, these findings should be interpreted as preliminary real-world observations.

## 1 Introduction

Neurodevelopmental disorders begin during childhood and encompass a wide clinical spectrum affecting an individual's cognitive, social, motor, and language development. These disorders include Attention-Deficit/Hyperactivity Disorder (ADHD), Autism Spectrum Disorder (ASD), Developmental Coordination Disorder, Learning Disabilities, Intellectual Disabilities, and Tourette Syndrome (1). The management of symptoms in individuals with neurodevelopmental disorders is increasingly relying on both pharmacological and non-pharmacological interventions (2).

Among neurodevelopmental disorders, ADHD is one of the most common childhood-onset conditions, with an estimated worldwide prevalence of approximately 5% in children and adolescents (3). ADHD is frequently associated with academic, social, emotional, and family-related impairments, and clinical management may become more challenging in the presence of comorbid neurodevelopmental or psychiatric conditions (4).

Psychostimulants are recommended as first-line pharmacological treatments for ADHD in children and adolescents (4). However, some patients show an inadequate response, experience clinically significant adverse effects, or cannot tolerate stimulant treatment. Appetite suppression, sleep disturbance, emotional lability, tics, and cardiovascular concerns may limit stimulant use in selected patients (4). Treatment may also be more challenging in the presence of comorbid autism spectrum disorder, intellectual disability, irritability, oppositional symptoms, or sleep problems. Therefore, non-stimulant medications may be required as monotherapy or adjunctive treatment in patients with partial response, poor tolerability, or complex comorbidity profiles. Guanfacine extended-release (GXR), an alpha-2A adrenergic receptor agonist, is one of the non-stimulant treatment options used either alone or in combination with psychostimulants (5–7).

Randomized controlled trials have demonstrated reductions in hyperactivity, impulsivity, and oppositional symptoms in children with ADHD symptoms and comorbid ASD (8, 9). Preliminary evidence for guanfacine more broadly also suggests potential benefits in selected patients with ADHD accompanied by tic disorders or other psychiatric comorbidities; however, evidence outside the core ADHD indication remains limited (10–12).

Children and adolescents with ADHD may also present with chronic medical conditions, making medication tolerability and potential interactions clinically relevant. GXR may represent an alternative or adjunctive treatment option in selected clinically complex cases. Common treatment-emergent adverse effects associated with GXR include somnolence, fatigue, sedation, dizziness, irritability, abdominal pain, and nausea (13, 14). Somnolence, fatigue, irritability, and appetite-related adverse effects have also been reported in clinical trials (8).

GXR is frequently used in combination with other psychotropic medications in routine clinical practice. Previous studies have demonstrated clinical benefits when GXR is combined with psychostimulants, including methylphenidate formulations and lisdexamfetamine (15). GXR's long-term effects, side effect profile in various patient groups, and individual dose-response variations, however, are still unclear (5). In particular, assessing data from actual clinical practice is essential to comprehending the medication's efficacy and dependability.

Although GXR has established efficacy in the treatment of ADHD, real-world data on its use in diagnostically heterogeneous children and adolescents with ADHD, including those with neurodevelopmental, psychiatric, cognitive, and medical comorbidities, remain limited. In particular, its tolerability and associated clinical changes in patients with comorbid ASD, intellectual disability, learning disorders, and concomitant psychotropic medication use warrant further investigation. Therefore, this study aimed to retrospectively examine clinical scale changes and tolerability patterns following GXR initiation in a sample of child and adolescent psychiatry patients.

## 2 Materials and methods

This study included 38 children and adolescents aged 6–18 years who were followed in a child and adolescent psychiatry clinic and were prescribed GXR for ADHD, with or without comorbid neurodevelopmental or psychiatric conditions. All participants received the extended-release formulation; immediate-release guanfacine was not used. GXR was generally initiated at 1 mg/day and titrated according to clinical response and tolerability. Dose increases were considered in patients with insufficient clinical response, whereas dose reduction or discontinuation was considered in patients who developed clinically significant adverse effects. Because treatment was provided under routine clinical conditions, no standardized study-specific titration protocol or fixed target dose was used. Dose adjustments were individualized during follow-up. The final daily dose ranged from 1 to 6 mg/day, corresponding to 0.01–0.15 mg/kg/day.

The study was designed as a retrospective observational chart review with a pre–post comparison of clinical scale scores before and after GXR initiation. Clinical data were obtained retrospectively from patient medical records. The Turgay DSM-IV-Based ADHD and Comorbid Disorders Screening and Rating Scale, Childhood Autism Rating Scale (CARS), Global Assessment Scale (GAS), and Clinical Global Impressions Scale-Severity (CGI-S) were routinely used during clinical follow-up. The Barkley Stimulant Side Effect Rating Scale was reviewed to evaluate methylphenidate-related adverse effects in patients receiving combination treatment. GXR-related adverse effects were identified through a review of clinical notes and follow-up records.

Inclusion criteria were being between 6 and 18 years of age, having a DSM-5 diagnosis of ADHD, and being prescribed GXR during routine clinical follow-up. Patients with available baseline data were included in the tolerability analysis. Patients who continued GXR for at least three months and had both baseline and follow-up scale assessments were included in the pre–post clinical outcome analyses. Patients with insufficient medical records or missing baseline clinical data were excluded. Baseline clinical scale scores were obtained immediately before or at the time of GXR initiation. Follow-up scale scores were obtained during routine clinical visits conducted between weeks 4 and 12 after GXR initiation. When more than one follow-up assessment was available within this interval, the assessment closest to week 12 was used in the pre–post analyses.

Concomitant psychotropic medications, including methylphenidate, antidepressants, antipsychotics, and mood stabilizers, were reviewed throughout the observation period. The names, doses, initiation status, and discontinuation status of these medications were obtained from the medical records. Concomitant psychotropic medication regimens and doses remained unchanged during the follow-up interval; only the GXR dose was adjusted according to clinical response and tolerability.

Clinical change was evaluated dimensionally using CGI-S, global functioning scores, and Turgay ADHD Scale scores. A categorical responder definition was not used as the primary outcome.

Psychiatric diagnoses were established according to DSM-5 criteria through routine clinical assessments and K-SADS-PL interviews. Psychiatric comorbidities were assessed using the Turkish version of the Kiddie Schedule for Affective Disorders and Schizophrenia for School-Age Children—Present and Lifetime Version (K-SADS-PL). Diagnostic evaluations were performed by child and adolescent psychiatry clinicians and supervised by senior child and adolescent psychiatrists. Data on cognitive functioning were obtained from patient files based on WISC-R or WISC-IV assessments that had previously been administered as part of routine clinical evaluations.

### 2.1 Ethical considerations

This study was conducted in accordance with the principles of the Declaration of Helsinki. Ethical approval was obtained from the Ege University Medical Research Ethics Committee with the approval number 25-1T/49. Written informed consent was obtained from the parents or legal guardians of the participants.

### 2.2 Data collection tools

#### 2.2.1 Sociodemographic data form

Sociodemographic data were extracted retrospectively from patient medical records, including age, sex, educational status, family history, comorbid medical conditions, medication use, and neurodevelopmental disorder history. Regular non-psychiatric medications were reviewed based on the available medical records, with particular attention to medications that could affect blood pressure, heart rate, sedation, or GXR metabolism. Medication names and treatment regimens were documented more consistently than exact dose information. The records were also reviewed for any initiation, discontinuation, or regimen change during the GXR observation period.

#### 2.2.2 Kiddie schedule for affective disorders and schizophrenia for school-age children—present and lifetime version (KSADS-PL)

The KSADS-PL is a semi-structured diagnostic interview developed by Kaufman et al. (16). The DSM-5–based Turkish version was adapted and validated by Ünal et al. (17). The first part of the schedule involves an unstructured interview covering the patient's and family's sociodemographic characteristics, the patient's complaints, developmental history, and general functionality. The second part includes over 200 specific symptom-based questions about the past and the last two months. The third part consists of evaluations developed to verify DSM-5 diagnoses. Information obtained from different sources is evaluated separately and then combined with the clinician's observational notes to be scored together (17).

#### 2.2.3 Turgay scale (Turgay DSM-IV-based ADHD and comorbid disorders scale)

Developed by Dr. Atilla Turgay and his team in 1995, this scale consists of three sections: Attention Deficit (I), Hyperactivity/Impulsivity (II), and features associated with ADHD (III). Sections 1 and 2 are based on DSM-IV criteria and contain a total of 18 items. Section 3 consists of 30 items assessing additional psychiatric symptoms. Responses are scored between 0 and 3, and scores of 2 or 3 are considered “positive” (18). The Turkish validity and reliability study was conducted by Ercan et al. (19).

#### 2.2.4 Clinical global impressions scale (CGI-S)

CGI-S scores range from 1 to 7, with higher scores indicating greater illness severity (20).

#### 2.2.5 Global assessment scale (GAS)

A scale used to assess functionality, with scores ranging from 0 to 100 (21). Scores of 61–100 indicate good functionality, 31–60 indicates moderate functionality, and below 30 indicates poor functionality.

#### 2.2.6 Childhood autism rating scale (CARS)

A 15-item scale developed by Schopler et al. to determine the diagnosis and severity of autism (22). A score above 30 indicates autism, with scores between 30 and 36 indicating mild to moderate autism, and above 37 indicating severe autism. The Turkish validity and reliability study was conducted by İncekaş Gassaloğlu et al. (23). Because CARS is an autism-specific instrument, analyses involving CARS scores were restricted to participants with comorbid ASD.

#### 2.2.7 Barkley stimulant side effect rating scale (BSSERS)

The BSSERS is a 17-item parent-rated scale developed to assess common adverse effects associated with stimulant medications. Each item is rated from 0 (absent) to 9 (severe), with higher scores indicating greater adverse-effect severity (24).

#### 2.2.8 Statistical analysis

The data's mean, standard deviation, median, minimum, maximum, frequency, and percentage values were presented as descriptive statistics. The Shapiro–Wilk test was used to examine the normality assumption of quantitative data. The Wilcoxon test was employed for variables that did not match the normality assumption, and the Paired-Sample *T*-test was used for those that did. Repeated Measures Analysis of Variance was performed to assess the difference between two time points on the Turgay ADHD Scale. IBM SPSS Statistics 25.0 software was used for all statistical analyses. For every analysis, a significance level of 0.05 was established. Because this was a retrospective chart review including all eligible patients treated during the study period, no *a priori* sample size calculation was performed. Therefore, the analyses should be considered exploratory. Bonferroni correction was applied to control for multiple comparisons across Turgay subscale analyses. Analyses involving CARS scores were performed only within the ASD subgroup.

## 3 Results

### 3.1 Sociodemographic findings and educational Status

This study presents information from 38 patients that satisfied the inclusion requirements. Male participants constituted 76.3% of the sample, whereas female participants accounted for 23.7%. With a mean age of 10.444 ± 2.697 years for girls and 12.069 ± 2.737 years for boys, the participants' average age is 11.684 ± 2.781 years. As for the participants' educational status, one case (2.6%) is in preschool, fourteen cases (36.8%) are in primary school, seventeen cases (44.7%) are in secondary school, and six cases (15.8%) are in high school. Thirty-one patients (81.6%) are receiving special education, whereas seven cases (18.4%) are not.

### 3.2 Clinical features

**Figure 1:**
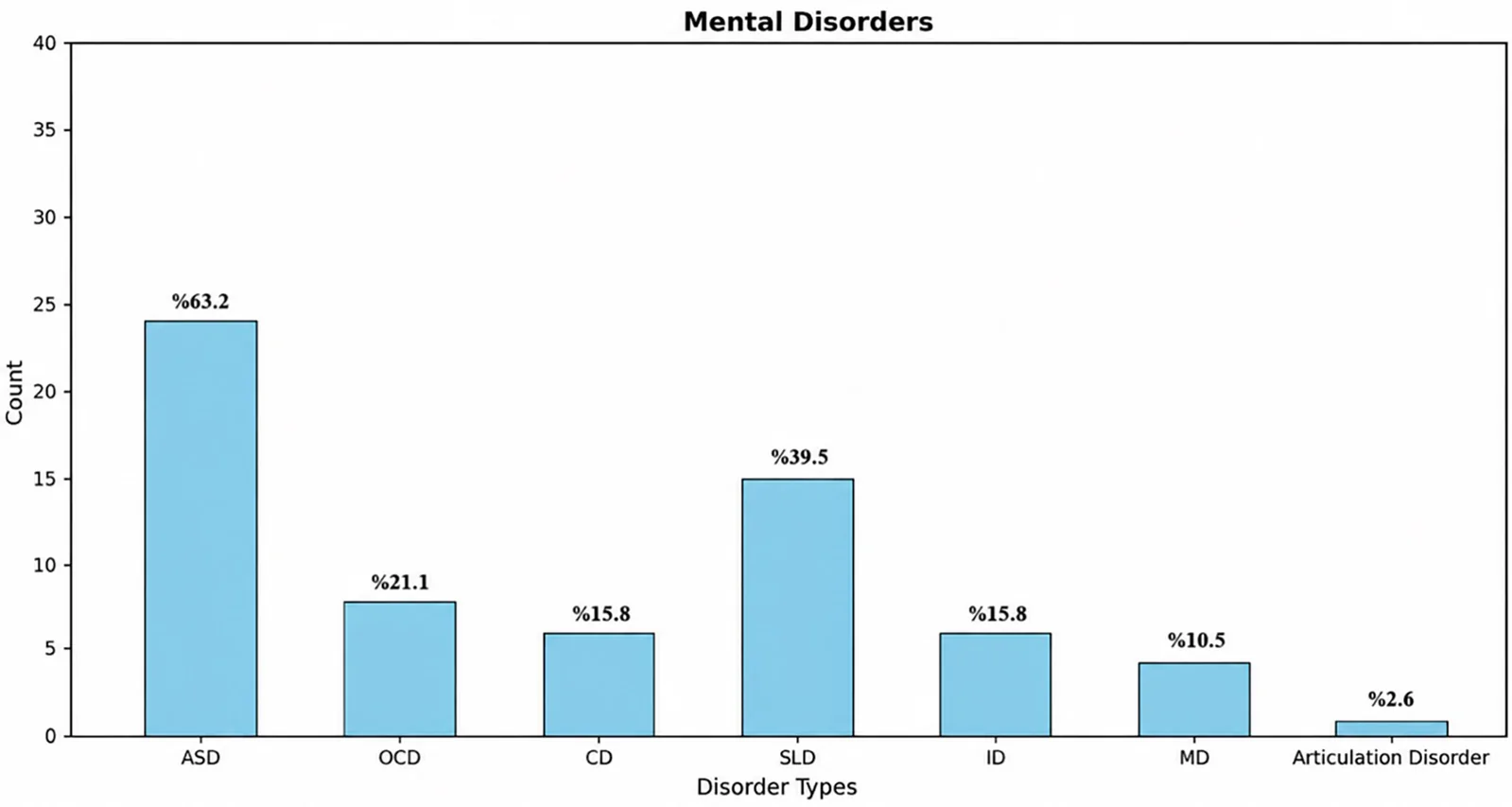
Distribution of psychiatric and neurodevelopmental comorbidities among participants with ADHD. ASD, autism spectrum disorder; SLD, specific learning disorder; ID, intellectual disability; OCD, obsessive-compulsive disorder; MD, mood disorder; CD, conduct disorder. All participants had ADHD. Comorbidity categories were not mutually exclusive because individual patients could have more than one accompanying diagnosis. Articulation disorder is shown without abbreviation to avoid confusion with attention deficit.

Figure 1 presents information on the cases' clinical condition. All participants had a diagnosis of ADHD. Comorbid neurodevelopmental and psychiatric conditions were common: 63.2% had ASD, 39.5% had specific learning disorder, 21.1% had OCD, and 15.8% had intellectual disability. Individual patients could have more than one comorbid diagnosis; therefore, the diagnostic categories were not mutually exclusive and their percentages did not sum to 100%. No participant had a psychotic disorder. All participants were receiving psychotropic medication. According to the most recent intelligence assessments, 10.5% of the participants demonstrated superior intelligence, 13.5% high intelligence, 7.9% borderline intellectual functioning, 10.5% mild intellectual disability, 7.9% moderate intellectual disability, and 50% exhibited intelligence within the normal range.

Twelve patients (31.6%) had chronic medical conditions, including epilepsy in six patients and thalassemia, hydrocephalus, Hashimoto's thyroiditis, Perthes disease, gastritis, and type 1 diabetes in one patient each. All 12 patients with chronic medical conditions were receiving regular non-psychiatric medications. These medications included antiepileptic agents, insulin therapy, thyroid hormone replacement, and gastroprotective medications, as documented in the medical records. Medication names and treatment regimens were available, although exact dose information was not consistently documented for all medications. These medications were reviewed with particular attention to their potential effects on blood pressure, heart rate, sedation, and GXR metabolism. No initiation, discontinuation, or change in the recorded non-psychiatric medication regimens was documented during the GXR observation period, and no clinically significant interaction with GXR was documented in the medical records.

### 3.3 Clinical profile of GXR administration

**Figure 2:**
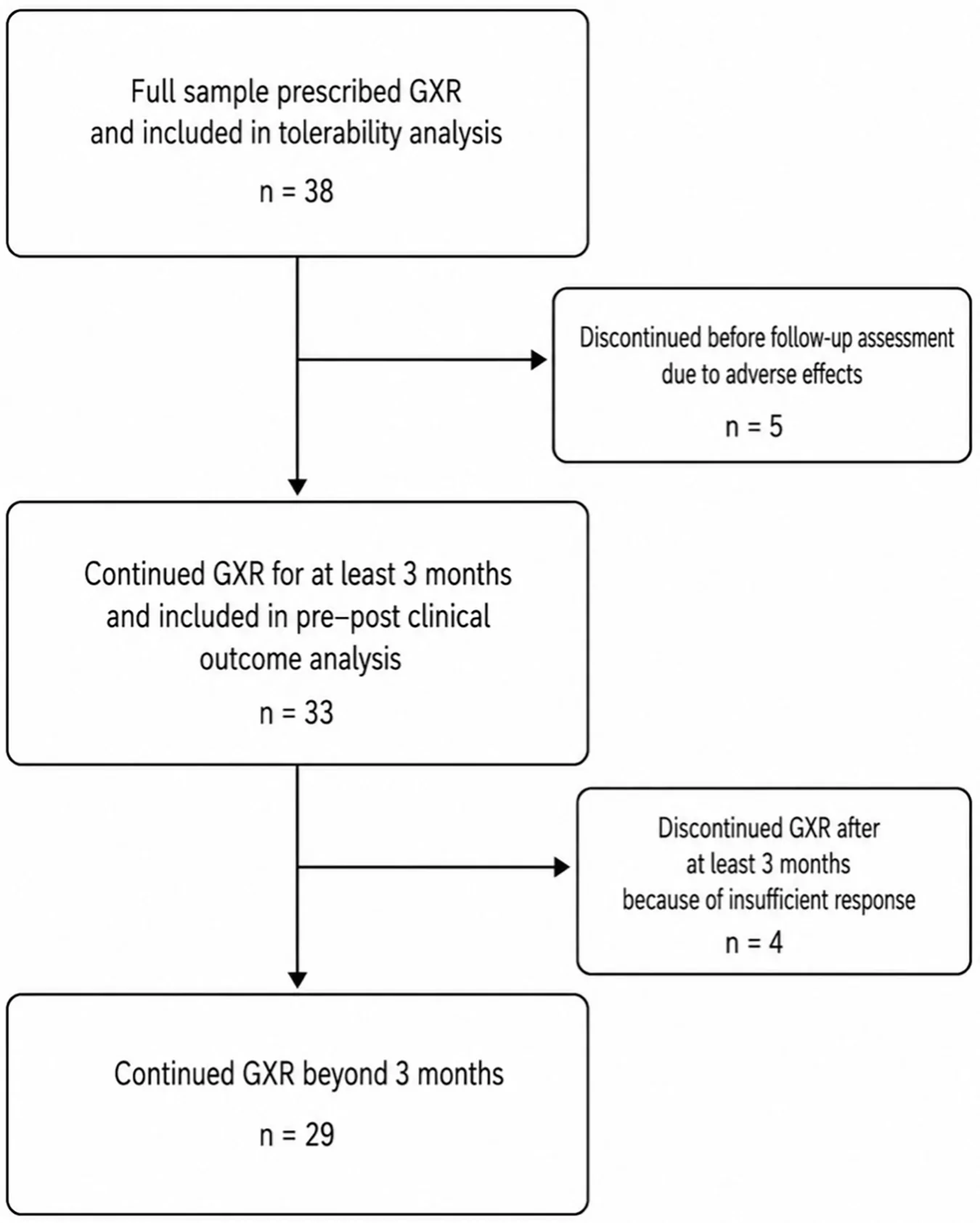
Flowchart of patient inclusion and analysis groups. The tolerability analysis included all 38 patients. The pre–post clinical outcome analyses included 33 patients who continued GXR for at least three months and had follow-up scale assessments available between weeks 4 and 12 after GXR initiation.

Table 1 summarizes GXR dosing and tolerability, together with psychotropic medication regimens documented at the most recent available clinical assessment. Because some patients had discontinued GXR by the time of their most recent assessment, not all medication regimens shown in the table included GXR. During the GXR follow-up interval, concomitant psychotropic medications and their doses remained stable, and only the GXR dose was adjusted according to clinical response and tolerability. Most patients received GXR as part of a combination psychotropic treatment regimen that included methylphenidate, SSRIs, antipsychotics, or mood stabilizers. Of the 38 patients prescribed GXR, five (13.2%) discontinued treatment before the follow-up assessment because of intolerable adverse effects. The remaining 33 patients (86.8%) continued GXR for at least three months and were included in the pre–post clinical outcome analyses. Subsequently, four of these 33 patients (12.1%) discontinued GXR because of insufficient clinical response, whereas 29 continued GXR beyond three months. GXR-related adverse effects were documented in 11 patients (28.9%) (Figure 2).

**Table 1:** GXR dosing and tolerability in the full sample and psychotropic medication regimens at the most recent clinical assessment (*n* = 38).

Variable	Mean ± SD	Min–Max	*n* (%)
Age at GXR initiation	10.73 ± 2.87	6–16	
GXR dose, mg/day	2.57 ± 1.05	1–6	
GXR dose, mg/kg/day	0.05 ± 0.03	0.01–0.15	
Psychotropic medication regimen at the most recent clinical assessment			
GXR			1 (2.6)
GXR + methylphenidate + SSRI			1 (2.6)
GXR + methylphenidate + AP			7 (18.4)
GXR + SSRI + AP			6 (15.8)
GXR + methylphenidate + SSRI + AP			5 (13.2)
GXR + methylphenidate + AP + MS			1 (2.6)
GXR + AP			8 (21.1)
AP			3 (7.9)
Methylphenidate + SSRI			1 (2.6)
Methylphenidate + AP			1 (2.6)
Methylphenidate + SSRI + AP			2 (5.3)
AP + MS			2 (5.3)
GXR-related adverse effect			
Present			11 (28.9)
Absent			27 (71.1)

SSRI, selective serotonin reuptake inhibitor; AP, antipsychotic; MS, mood stabilizer.

Restlessness was the most frequently reported GXR-related adverse effect (*n* = 6, 15.8%), followed by dizziness (*n* = 3, 7.9%), somnolence (*n* = 2, 5.3%), and nightmares (*n* = 2, 5.3%). Anxiety, fatigue, dry mouth, diarrhea, and urinary incontinence were each reported in one patient (2.6%). Among patients who discontinued GXR because of adverse effects, restlessness was the most common reason for discontinuation. Three of the five patients who discontinued GXR because of adverse effects had comorbid ASD, and two had intellectual disability. Among these five patients, one had ADHD without a documented comorbid neurodevelopmental or psychiatric disorder, one had ADHD and ASD, one had ADHD, ASD, and ID, one had ADHD, ASD, ID, language disorder, and mood disorder, and one had ADHD and OCD. One patient who discontinued GXR because of adverse effects had a chronic medical condition, namely hydrocephalus. Within the ASD subgroup (*n* = 24), baseline CARS scores were not significantly associated with GXR-related adverse-effect status. Exploratory analyses were also conducted to examine potential factors associated with GXR-related adverse effects. The frequency of GXR-related adverse effects was 33.3% in patients with comorbid ASD and 21.4% in patients without ASD; however, this difference was not statistically significant (Fisher's exact test, *p* = .488). Among patients with comorbid ASD, the most commonly reported adverse effect was restlessness (12.5%), followed by somnolence (8.3%) and orthostatic dizziness (8.3%). Fatigue, nightmares, and urinary incontinence were each reported in 4.2% of patients with comorbid ASD. The frequency of GXR-related adverse effects was 33.3% in patients with chronic physical illness and 26.9% in those without chronic physical illness, with no statistically significant difference between groups (Fisher's exact test, *p* = .714). These findings should be interpreted cautiously because of the small sample size and retrospective nature of adverse-effect documentation.

### 3.4 Symptom management with GXR and changes in functionality

Among the 33 patients with follow-up assessments obtained between weeks 4 and 12 after GXR initiation, the median CGI-S score decreased from 5 to 3, and this difference was statistically significant (*p* < .001). The median GAS score increased from 55 to 75, and this difference was also statistically significant (*p* < .001) (Table 2).

**Table 2:** Comparison of CGI-S and global functioning scores before and after GXR initiation among patients with follow-up assessments obtained between weeks 4 and 12 (*n* = 33).

Outcome measure	Before	After	Test statistic	*p*
	Median (min–max)	Median (min–max)		
CGI-S	5 (3–7)	3 (2–5)	−4.827	**<0.001** **
GAS	55 (35–75)	75 (35–95)	−4.676	**<0.001** **

Bold values indicate statistical significance at *p* < 0.05.

** Wilcoxon test.

CGI-S, clinical global impression-severity; GAS, global assessment scale.

No significant association was found between change in CGI-S scores and age or sex. Within the ASD subgroup, baseline CARS scores were not significantly associated with change in CGI-S scores. An exploratory weak association was observed between final weight-adjusted GXR dose and change in CGI-S score (*r* = −0.349, *p* = .040). Because dose titration was clinically determined rather than randomized, this finding should not be interpreted as evidence of a causal dose–response relationship. When baseline CGI-S score was considered, a moderate negative correlation was observed with CGI-S change (Spearman's rho = −0.417, *p* = .018).

Weight-adjusted GXR dose was entered as the independent variable, and change in CGI-S score was entered as the dependent variable in the linear regression model. The model explained a limited proportion of variance in CGI-S change (*R*^2^ = 0.144); therefore, this finding should be considered exploratory.

Parents of 33 patients completed the Turgay ADHD Scale at baseline and during follow-up between weeks 4 and 12 after GXR initiation. The number of symptoms graded 2 and 3 as well as the overall scale score were compared. Regarding the overall scale scores, the addition of GXR resulted in notable reductions in the subcategories of oppositional defiant disorder (ODD), hyperactivity and impulsivity (HI), attention deficit (AD), and conduct disorder-related symptoms (CD) (Table 3). There were no gender-specific variations in the changes in total scale scores.

**Table 3:** Changes in Turgay scale total scores before and after GXR initiation among patients with follow-up assessments obtained between weeks 4 and 12 (*n* = 33).

Turgay subscale total score	Before	After	Test statistic	*p*
	Mean ± SD	Mean ± SD		
AD total	18.636 ± 6.009	10.303 ± 4.319	8.360	**<0** **.** **001** *
HI total	14.09 ± 7.863	7.666 ± 5.549	−4.792	**<0**.001**
ODD total	7.181 ± 7.443	3.303 ± 4.111	−3.923	**<0**.001**
CD total	2.09 ± 4.791	0.878 ± 2.57	−2.371	0.018**

Bold values indicate statistical significance at *p* < 0.0125.

* Paired samples *t*-test, **Wilcoxon test.

AD, attention deficit; HI, hyperactivity/impulsivity; ODD, oppositional defiant disorder; CD, conduct disorder.

Following the initiation of GXR treatment, significant reductions were also observed in the subdomains of attention deficit (AD), hyperactivity/impulsivity (HI), oppositional defiant disorder (ODD), and conduct disorder-related symptoms (CD) based on the total number of symptoms reported on the scale (Table 4). After correction for multiple comparisons, reductions in attention deficit, hyperactivity/impulsivity, and oppositional defiant symptoms remained significant, whereas changes in conduct disorder-related symptoms should be interpreted cautiously. There were no gender-specific variations in the changes in symptom scores.

**Table 4:** Changes in Turgay scale symptom counts before and after GXR initiation among patients with follow-up assessments obtained between weeks 4 and 12 (*n* = 33).

Turgay subscale symptom count	Before	After	Test statistic	*p*
	Mean ± SD	Mean ± SD		
AD symptoms	6.848 ± 1.769	4.03 ± 1.878	−4.805	<0.001**
HI symptoms	5.105 ± 2.845	3.5 ± 2.479	−4.752	**<0**.001*
ODD symptoms	2.424 ± 2.634	1.333 ± 1.881	−3.443	**0**.001**
CD symptoms	0.636 ± 1.453	0.333 ± 0.924	−2.06	0.039**

Bold values indicate statistical significance at *p* < 0.0125.

* Paired samples *t*-test, **Wilcoxon test.

AD, attention deficit; HI, hyperactivity/impulsivity; ODD, oppositional defiant disorder; CD, conduct disorder.

The differences in the changes in the AD, HI, ODD, and CD subcategories were investigated using a Repeated Measures Analysis of Variance (ANOVA). Both the total scale scores and the number of symptoms showed statistically significant differences (*p* < 0.001). The order of changes in the whole scale score was AD > HI > ODD > CD, according to *post hoc* comparisons (*F* = 19.37, *p* < 0.001, *ηp*^2^ = 0.377). The order of changes for the number of symptoms was AD > HI > ODD > CD (F = 26.53, *p* < 0.001, *ηp*^2^ = 0.453).

## 4 Discussion

This retrospective chart review provides clinical observations on GXR use in a diagnostically heterogeneous sample of children and adolescents with ADHD, with or without comorbid neurodevelopmental or psychiatric conditions. The findings suggest that GXR use was associated with improvements in ADHD symptom ratings and global functioning scores during follow-up.

First, it is noted that the percentage of male participants (76.3%) is substantially greater than the percentage of female participants (23.7%). According to a number of studies in the literature, men are more likely than women to suffer from neurodevelopmental problems (25, 26).

Changes in global functioning were assessed using CGI-S and GAS scores. Follow-up CGI-S and GAS scores obtained between weeks 4 and 12 after GXR initiation showed significant improvements compared with baseline (*p* < .001). Previous studies have suggested that GXR may reduce ADHD symptom severity, particularly hyperactivity/impulsivity and oppositional symptoms (27). These findings are consistent with previous studies suggesting that GXR may be associated with improvements in ADHD symptoms and global functioning. A weak association was observed between final weight-adjusted GXR dose and change in CGI-S score. However, this finding should not be interpreted as demonstrating a causal dose–response effect. In routine clinical practice, patients with greater baseline severity, persistent symptoms, partial response, or better medication tolerability may be titrated to higher doses. Therefore, the observed association may reflect confounding by indication or severity. Prospective studies using standardized titration schedules are required to clarify whether clinical outcomes differ according to GXR dose.

However, these findings should be interpreted cautiously because most patients were receiving concomitant psychotropic medications, including methylphenidate, SSRIs, antipsychotics, or mood stabilizers. Although these medications and their doses remained stable during the follow-up interval and only the GXR dose was adjusted, their ongoing therapeutic effects could not be separated from the potential contribution of GXR. Therefore, the observed clinical improvements cannot be attributed specifically or solely to GXR.

Significant reductions in inattention, hyperactivity/impulsivity, and oppositional defiant symptom scores were observed following GXR initiation. These findings are consistent with previous evidence suggesting that GXR may be associated with reductions in ADHD symptom severity (28).

Comorbid ASD was common in this clinically complex sample and was present in 63.2% of participants. This high rate likely reflects the selected and diagnostically heterogeneous nature of the study population and should not be interpreted as an estimate of ASD prevalence among all children and adolescents with ADHD. GXR may be considered a potential treatment option in selected patients with ADHD and comorbid ASD, with careful monitoring for adverse effects. This interpretation should be made cautiously because of the retrospective design, small sample size, and lack of a control group. This study did not aim to evaluate the effect of GXR on core ASD symptoms, and autism-related measures were used primarily to characterize clinical features rather than to define treatment response. Analyses involving CARS scores were restricted to the ASD subgroup.

### 4.1 GXR Side effect profile

Restlessness (15.8%), dizziness (7.9%), somnolence (5.3%), nightmares (5.3%), dry mouth (2.6%), and fatigue (2.6%) were the most commonly reported adverse effects after GXR use. Previous clinical trials have reported somnolence, fatigue, sedation, dizziness, and blood-pressure reductions during GXR treatment (13, 29). In exploratory subgroup analyses, the frequency of GXR-related adverse effects did not differ significantly according to the presence of comorbid ASD or chronic physical illness. Although adverse effects were numerically more frequent in patients with ASD and in those with chronic physical illness, these differences were not statistically significant and should be interpreted cautiously because of the small sample size. Notably, a considerable proportion of participants who were unable to tolerate the medication had a diagnosis of autism spectrum disorder (ASD). Previous studies suggest that individuals with ASD may show increased sensitivity to psychotropic medications and adverse effects (30). These findings suggest that careful dose titration and close monitoring may be particularly important when prescribing GXR to patients with ASD**.** It is known that individuals with intellectual disabilities also exhibit neurological, pharmacokinetic, and psychological differences, which lead to increased sensitivity to psychotropic drugs and variable pharmacodynamic responses (31). Our findings suggest that GXR may be tolerated by some patients with comorbid intellectual disability; however, this observation should be interpreted cautiously because of the small number of cases.

### 4.2 Clinical implications and strengths of the study

This study provides real-world clinical data on GXR use in a diagnostically heterogeneous group of children and adolescents with ADHD and frequent neurodevelopmental, psychiatric, cognitive, and medical comorbidities. An important strength is that clinical change was observed at both the symptom and functional levels. Improvements were documented not only in ADHD-related symptom ratings but also in global illness severity and functioning. In addition, outcomes were assessed using several complementary and validated clinical instruments, including CGI-S, GAS, and the Turgay ADHD Scale, rather than relying on a single outcome measure. The inclusion of tolerability data and clinical discontinuation patterns provides additional information relevant to routine practice.

### 4.3 Limitations

This study has several limitations. First, its retrospective observational design and the absence of a control group preclude causal inference. Second, the sample size was small and the age range was broad, limiting statistical power and generalizability. Third, the sample was diagnostically heterogeneous, and nearly all participants were receiving concomitant psychotropic medications. Therefore, the effects of concurrent treatments, natural symptom fluctuation, clinician expectations, regression to the mean, and other unmeasured clinical factors cannot be separated from the potential contribution of GXR. Accordingly, the observed improvements cannot be attributed specifically or exclusively to GXR.

Dose titration was clinically determined rather than randomized or standardized. Patients with greater symptom severity, partial response, or better tolerability may have received higher doses, creating the possibility of confounding by indication and severity. Baseline and follow-up assessments were obtained from routine clinical records. Follow-up assessments were conducted within a variable interval of 4–12 weeks after GXR initiation rather than at a fixed study-mandated time point, which may have introduced variability into the pre–post comparisons. Adverse effects were identified through chart review rather than a structured GXR-specific adverse-effect scale, and information regarding their severity, duration, and management was limited. Analyses involving CARS were restricted to the ASD subgroup and should not be generalized to the full sample. Although regular non-psychiatric medication names and treatment regimens were available in the medical records, exact dose information was not consistently documented for all medications, and intermittent or over-the-counter medication use may not have been fully captured. Missing data, documentation bias, multiple statistical comparisons, and limited power of subgroup and regression analyses also require cautious interpretation. Prospective controlled studies with larger samples, standardized titration and assessment schedules, and stable concomitant treatment regimens are needed.

## 5 Conclusion

In this retrospective chart review, GXR use was associated with improvements in ADHD symptom ratings and global functioning in a diagnostically heterogeneous sample of children and adolescents with ADHD. However, adverse effects were documented in a clinically relevant proportion of participants and resulted in treatment discontinuation in some cases. Because the study was retrospective, uncontrolled, small, and included extensive concomitant psychotropic medication use, the observed improvements cannot be attributed solely to GXR. These findings should therefore be considered preliminary and hypothesis-generating. Larger prospective controlled studies with standardized titration schedules, assessment intervals, and stable concomitant treatment regimens are needed to clarify the effectiveness and tolerability of GXR in clinically complex populations.

## Data Availability

The raw data supporting the conclusions of this article will be made available by the authors, without undue reservation.
